# Effectiveness of a web-based screening and brief intervention with weekly text-message-initiated individualised prompts for reducing risky alcohol use among teenagers: study protocol of a randomised controlled trial within the ProHEAD consortium

**DOI:** 10.1186/s13063-018-3160-z

**Published:** 2019-01-22

**Authors:** Silke Diestelkamp, Lutz Wartberg, Michael Kaess, Stephanie Bauer, Christine Rummel-Kluge, Katja Becker, Heike Eschenbeck, Hans-Joachim Salize, Markus Moessner, Christiane Baldus, Nicolas Arnaud, Rainer Thomasius, the ProHEAD consortium, Michael Kaess, Stephanie Bauer, Rainer Thomasius, Christine Rummel-Kluge, Heike Eschenbeck, Hans-Joachim Salize, Katja Becker, Katja Bertsch, Sally Bilic, Romuald Brunner, Johannes Feldhege, Christina Gallinat, Sabine C. Herpertz, Julian Koenig, Sophia Lustig, Markus Moessner, Fikret Özer, Peter Parzer, Franz Resch, Sabrina Ritter, Jens Spinner, Silke Diestelkamp, Kristina Wille, Sabrina Baldofski, Elisabeth Kohls, Lina-Jolien Peter, Vera Gillé, Hanna Hofmann, Laya Lehner, Elke Voss, Jens Pfeiffer, Alisa Samel

**Affiliations:** 1 University Hospital Hamburg-Eppendorf, German Center for Addiction Research in Childhood and Adolescence, Martinistr. 52, W29, 20246 Hamburg, Germany; 2 Clinic of Child and Adolescent Psychiatry, Centre of Psychosocial Medicine, Heidelberg University Hospital, Blumenstraße 8, 69115 Heidelberg, Germany; 3 University Hospital of Child and Adolescent Psychiatry and Psychotherapy, University of Bern, Bolligenstrasse 111, Stöckli, 3000 Bern 60, Switzerland; 4 Centre for Psychotherapy Research, University Hospital Heidelberg, Bergheimer Straße 54, 69115 Heidelberg, Germany; 5 Clinic and Policlinic of Psychiatry and Psychotherapy, University of Leipzig, Semmelweisstraße 10, 04103 Leipzig, Germany; 6 Department of Child and Adolescent Psychiatry, Psychosomatics and Psychotherapy, Philipps-University of Marburg, Hans-Sachs-Str. 6, 35039 Marburg, Germany; 7 Department of Psychology, University of Education Schwäbisch Gmünd, Oberbettringer Str. 200, 73525 Schwäbisch Gmünd, Germany; 8 Mental Health Services Research Group, Central Institute of Mental Health, Medical Faculty Mannheim /Heidelberg University, Germany, J5, 68159 Mannheim, Germany

**Keywords:** Adolescence, Binge drinking, Web-based intervention, Text-message intervention, Booster, ProHEAD

## Abstract

**Background:**

Early and excessive alcohol use is a significant threat to healthy development. Evidence supports the effectiveness of electronic alcohol interventions for young drinkers. However, effects are typically small and studies targeting under 18-year-olds are scarce. This trial is the first to evaluate the effectiveness of a single-session, brief, motivational, web-based intervention (ProWISE) plus weekly text-message-initiated individualised prompts (TIPs) in reducing alcohol consumption and alcohol-related harm among children and adolescents aged ≥ 12 years. TIPs are designed to decrease risky alcohol use by reaching youth in the contexts of their everyday lives and by providing individualised feedback on drinking intentions, actual drinking and succession in achieving personal goals for low-risk drinking or abstinence.

**Methods/Design:**

The trial is part of the multicentre consortium ProHEAD testing e-interventions for mental health problems in children and adolescents. Participants in grades 6–13 aged ≥ 12 years will be recruited in schools which participate in ProHEAD (target *N* = 15,000). Main criterion for inclusion in the ProWISE-TIP trial is a positive screening for at-risk alcohol use in the CRAFFT-d questionnaire (target *n* = 1076). In a multicentre, four-arm, randomised controlled design the following groups will be compared: (A) web-based intervention plus TIPs for 12 weeks; (B) web-based intervention plus text-message-initiated assessment of alcohol consumption for 12 weeks; (C) web-based intervention only; and (D) alcohol-related psychoeducation. TIPs will be delivered shortly before and after high-risk situations for excessive alcohol use and will be tailored to age, gender, drinking motives and alcohol consumption. Study participants will be followed up at three, six and nine months in the ProWISE-TIP trial and at one and two years in the ProHEAD consortium. Primary outcome is alcohol use in the past 30 days at nine months after enrolment. Secondary outcomes are alcohol-related problems, co-occurring substance use, health service utilisation, mental health problems and quality of life.

**Discussion:**

Trial results will generate important evidence on how to enhance effectiveness of single-session, web-based alcohol interventions for youth. The ProWISE-TIP intervention, if effective, can be used as a stand-alone alcohol intervention or as an add-on to school-based or community-based alcohol prevention programs.

**Trial registration:**

German Clinical Trials Register, DRKS00014606 Registered on 20 April 2018.

**Electronic supplementary material:**

The online version of this article (10.1186/s13063-018-3160-z) contains supplementary material, which is available to authorized users.

## Background

Early and excessive alcohol use in adolescence is a significant threat to healthy development and a major public health concern. According to the Global Burden of Disease Study, alcohol use is among the top three risk factors contributing to the worldwide burden of disease [1]. While overall alcohol use by children and adolescents in Germany has decreased in the past 15 years [2], binge drinking, i.e. the consumption of five (four for girls) or more alcoholic beverages at one drinking occasion, is prevalent among 16.7% and 11.4% of male and female 12- to 17-year-olds, respectively, in the past 30 days [3]. Among 16- to 17-year-old boys, 37.1% report binge drinking in the past month (26.7% among girls) [3], indicating that a substantial proportion of the young population is at risk for experiencing short- and long-term negative consequences of risky alcohol use. Even heavier alcohol consumption patterns are common in German youth. In a state-wide representative sample, 5.6% of adolescents reported problematic alcohol use [4]; in a nationwide representative sample, the prevalence of problem drinking was 5.0% (girls: 5.1%, boys: 5.0%) in 12- to 17-year-olds [5]. Furthermore, in 2016 a total of 23,627 patients aged ≤ 19 years were admitted to German hospitals with an ICD-10 F10 diagnosis of Mental and Behavioural Disorders due to Use of Alcohol [6]. Among the 15- to 20-year-old inpatients, this diagnosis was the second most frequent reason for hospitalisation. Other short-term health risks associated with risky alcohol use for children and adolescents include aggressive and risky sexual behaviour, as well as elevated rates of injury and traffic accidents [7]. Moreover, heavy episodic drinking in adolescence is associated with a number of social and developmental problems, such as social conflicts, delinquency and problems of academic adjustment [8, 9], which also put children and adolescents at risk for chronification of problematic substance use patterns into adulthood [10]. Beyond these personal risks, alcohol-related problems also impose significant economic burden on public healthcare [11].

Thus, early recognition and indicated preventive interventions are needed to tackle risky alcohol use in childhood and adolescence. Acknowledging this, the American Academy of Pediatrics has published a policy statement with the recommendation to introduce substance use screening, brief intervention and referral to treatment (SBIRT) in all clinical settings serving paediatric populations [12]. In Germany, however, despite recommendation in the S3-Guideline for treatment of alcohol-related disorders in children and adolescents [13], alcohol SBIRT for children and adolescents is not part of standard primary care [2, 6]. Early detection of and intervention for risky alcohol use is especially challenging among youth, since members of this age group typically show the smallest rates of access to the help system [14–16].

Web-based interventions have been increasingly acknowledged in their capacity to lessen existing barriers for contacting the help system, particularly for at-risk populations [17, 18]. Evidence of such interventions to foster positive behaviour change, symptom reduction and improvement of health status is growing for a range of behavioural and mental health problems, including screening, prevention and early intervention for problematic substance use [19, 20]. Evidence indicates that fully automated brief motivational interventions are feasible and well accepted [21] and have the potential to reduce drinking and related harms for emerging adult at-risk drinkers up to 12 months after the intervention [22] with typically small but consistent effect sizes comparable to more conventional health professional-delivered interventions for substance use outcomes [23]. In a previous study, our research group developed a fully automated, single-session, web-based, brief motivational intervention targeting at-risk substance-using children and adolescents (aged 16–18 years), which was tested in four European countries [24, 25]. Compared to the assessment-only control group, the intervention was effective in decreasing past-month drinking as assessed by an AUDIT-C-based index score for drinking frequency, quantity and frequency of binge drinking at the three-month follow-up. However, results were limited by a high drop-out rate at the three-month follow-up (85.5%). In sum, aggregated evidence [17, 26] supports the utilisation of electronic alcohol interventions in reducing alcohol consumption and related harms in populations of young drinkers, but effect sizes tend to be small and different hypothesis have been tested as to how to increase effects of web-based alcohol interventions.

User engagement (i.e. the degree to which users find, access and actually use intervention content) is crucial for digital intervention programs, especially when they are designed as stand-alone interventions not supported by face-to-face interactions with a counsellor [27]. Designing user-accepted interventions requires tailoring of content, design and usability to the target groups preferences (user-centredness) [27]. User-centred interventions correspond with the habits and characteristics of the target population which should be reflected in the functional specifications of a behaviour change program. Failure in addressing user needs and characteristics adequately has been identified as a major barrier to uptake and impact of web-based interventions [28]. In addition to tailoring, self-monitoring, personalised feedback and reminders (‘prompts’) were found to be essential features enhancing user engagement as well as effectiveness of web-based alcohol interventions [17, 27].

In this trial, we will therefore test the effects of the introduction of highly individualised prompts (i.e. ‘messages, reminders, or brief feedback communicated to participants multiple times over the duration of an intervention’ [29]) to a web-based alcohol intervention. These prompts have the potential to reach participants in the context of their everyday lives as proposed by the ecological momentary intervention (EMI) approach [30]. Frequent contacts preceding high-risk situations seem especially appropriate for the target population of children and adolescents, because adolescence is a developmental period of increased proneness to risk-taking behaviour such as excessive alcohol use [31]. In this developmental phase, impulsive processes influence behaviour more strongly and self-regulatory processes have a smaller impact on behaviour than in adulthood. Additionally, self-regulatory processes presumably have a smaller impact on behaviour in high-risk situations, e.g. in situations with high peer pressure for drinking or under the influence of acute alcohol [32], so that interventions which aim at strengthening self-regulatory processes are presumed to have a stronger impact if delivered shortly before high-risk situations [30, 33].

A recent meta-analysis provided support for the effectiveness of prompts in increasing user engagement with digital interventions, especially when they were introduced shortly after completion of the intervention [34]. In particular, text-message-based prompts were found to increase user engagement and yield significant effects on an increased readiness to change drinking behaviour as well as reductions in heavy drinking [17]. Effectiveness of text-messaging interventions for adolescents’ and young adults’ substance use was additionally supported by a recent meta-analysis including 14 studies testing this approach [35]. Findings revealed that text-message-based interventions lead to substantial reductions in alcohol and tobacco use with a summary effect size of *d* = 0.25. Furthermore, a recent study with *n* = 765 at-risk alcohol consuming young adults (aged 18–25 years) found a weekly text-message-based assessment of drinking intentions and tailored feedback over a period of 12 weeks to be effective in reducing frequency of binge drinking, drinks per drinking day and prevalence of alcohol-related injury at nine-month follow-up [33]. In sum, evidence for the effectiveness of individualised prompts as an add-on to a web-based alcohol intervention is promising. However, the majority of evidence for young populations stems from studies with college students [31] and studies targeting under 18-year-olds are scarce [17, 19, 22, 29]. In the light of the early onset of alcohol misuse and addictive developmental trajectories [10], it is timely that the evidence base for this approach is broadened by testing it in the age group of under 18-year-olds.

### Aims

In this trial we will further develop a previously evaluated youth-specific, web-based, single-session, brief motivational alcohol intervention [24] to increase positive effects on reductions in alcohol consumption and alcohol-related harm in children and adolescents aged ≥ 12 years identified as risky drinkers. Specifically, we will integrate text-message-initiated individualised prompts (TIPs) following the initial delivery of the web-based, single-session, brief motivational intervention (ProWISE intervention) and test this version of the intervention in a randomised controlled trial (RCT). Main goals of this trial are: (1) development of a user-centred, time-efficient and youth-specific text-message-initiated technique for delivering highly individualised prompts assessing drinking intentions and actual alcohol use and providing individualised feedback; (2) pilot testing of feasibility and acceptability of the new intervention components (i.e. TIPs) and their refinement according to results from the pilot testing and evaluation of focus group interviews; and (3) evaluation of effectiveness of the TIPs as boosters for the ProWISE intervention in a four-arm randomised controlled design.

## Methods/Design

### Design

The ProWISE-TIP trial is a sub-project (SP) of the multisite, prospective ProHEAD consortium. The aim of ProHEAD is the development, implementation and evaluation of Internet-based programs promoting mental health in healthy children and adolescents, preventing mental health problems (depression, eating disorders, risky alcohol use) in those who are at high risk and enhancing help-seeking of children and adolescents with mental health problems [36]. The ProHEAD consortium aims to improve access to prevention and care for young people aged ≥ 12 years by capitalising on novel E-health tools. For a detailed description of the ProHEAD consortium design, please refer to Kaess et al. [36]; for details on the other SPs, please refer to the respective study protocols in this issue [37–39].

**Fig. 1:**
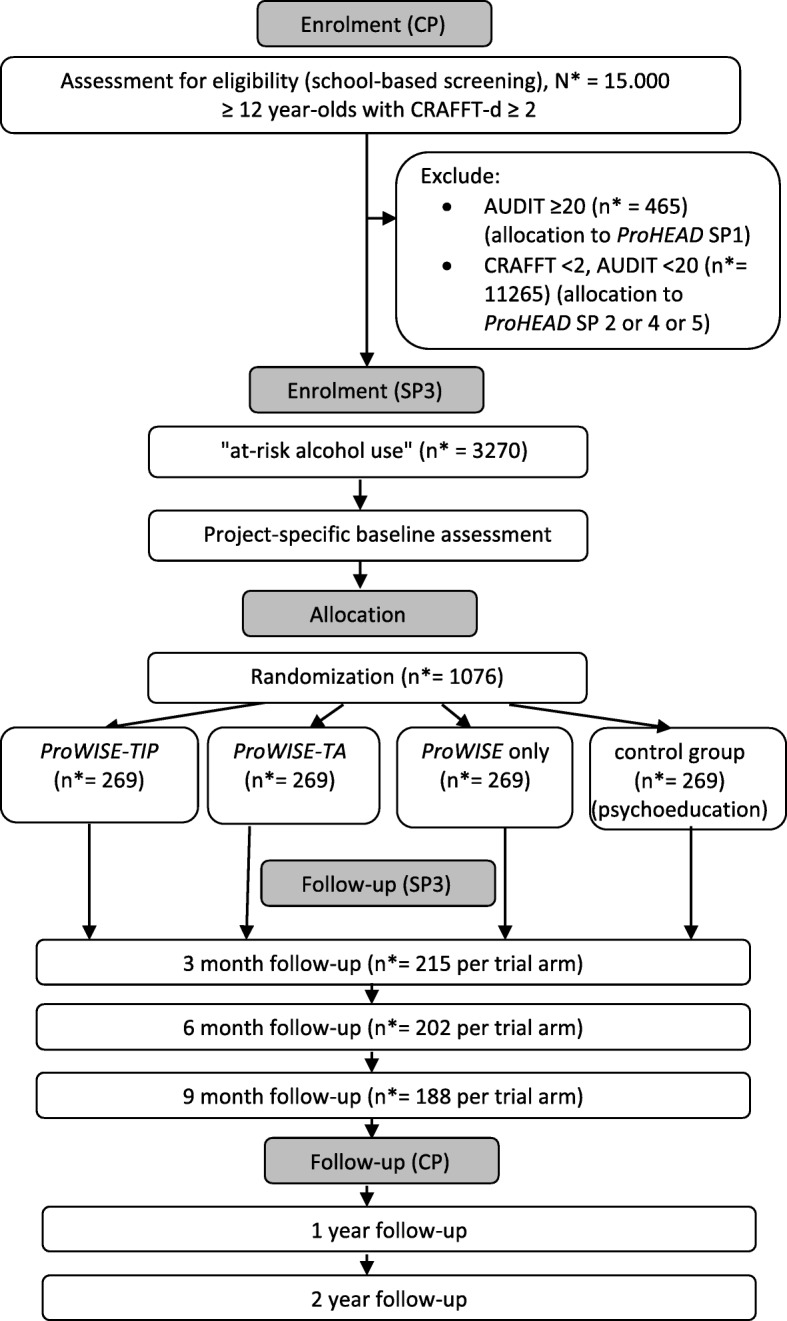
Trial flow diagram. *Note*: * refers to the proposed sample size, *TIP* text-message-based individualised prompt, *TA* text-message-based assessment, *CP* central project (ProHEAD project), *SP3* sub-project 3 (ProWISE-TIP trial)

Effectiveness of the ProWISE-TIP intervention will be tested in a four-armed, randomised controlled design with observer blinded group allocation with trial conditions: (A) ProWISE plus TIP for 12 weeks (ProWISE-TIP); (B) ProWISE plus text-message-initiated assessment (TA) for 12 weeks (ProWISE-TA); (C) ProWISE intervention only (ProWISE only); and (D) web-based psychoeducation on alcohol use in childhood and adolescence (control group). Participants will be followed up at three, six and nine months after enrolment in the SP and at one and two years after enrolment in the central project (ProHEAD consortium) (Fig. 1). A schedule of enrolment, assessment and intervention is provided in Fig. 3 and a populated Standard Protocol Items: Recommendations for Interventions Trials (SPIRIT) Checklist is provided in Additional file 1.

### Participants

The project will utilize a large-scale school-based sample of children and adolescents (*N* = 15,000 children and adolescents, grades 6–13, aged ≥ 12 years), recruited in five regions of Germany (Hamburg, Heidelberg, Leipzig, Marburg, Schwäbisch Gmünd). In the central project (ProHEAD consortium), online assessments of a broad variety of mental health problems and health-risk behaviours will be conducted at baseline and at two annual follow-ups. Following the baseline assessment, children and adolescents will be identified as either currently ‘healthy’, ‘high-risk’ (including subthreshold mental health problems and diverse health-risk behaviours) or having ‘mental health problems’ (which includes clinically relevant levels of psychopathology and/or indicators of threat to self and others). Based on these screening profiles, children and adolescents will be allocated to one of five programs according to their individual needs, i.e. increasing face-to-face mental help-seeking in children and adolescents with a screening result at baseline indicating mental health problems (SP1) [36], preventing eating disorder symptoms in children and adolescents with a screening result at baseline indicating a risk for the development of an eating disorder (SP2) [37], reducing alcohol misuse in children and adolescents with a screening result at baseline indicating risk for the development of alcohol misuse (SP3, ProWISE-TIP trial), preventing depressive symptoms in children and adolescents with a screening result at baseline indicating a risk for the development of depression (SP4) [38], promoting mental health and preventing mental health problems in children and adolescents with a screening result at baseline indicating no mental health problems (SP5) [39]. All programs provide online information as well as additional modules such as prompts or chat counselling with mental health professionals.

### Inclusion and exclusion criteria

Children and adolescents (1) in grades 6–13 aged ≥ 12 years, (2) with a positive screening result for at-risk alcohol use based on the CRAFFT-d screening tool (≥ 2) [40], (3) and written informed consent from both participant and legal guardian are eligible for inclusion in the ProWISE-TIP trial. Children and adolescents with (1) current alcohol use disorder according to the Alcohol Use Disorders Identification Test (AUDIT) (≥ 20) [41] or (2) current psychiatric disorders, (3) illiteracy, (4) no possession of a mobile phone, no access to the Internet or no sufficient German language skills will be excluded from participation in the ProWISE-TIP trial (Fig. 1). Children and adolescents with a total score of ≥ 20 in the AUDIT [41] or a total score of 20–40 points on the Strengths and Difficulties Questionnaire (SDQ) [42] or a score above the defined thresholds for one of its sub-scales: emotional symptoms (scores: 7–10), conduct problems (scores: 5–10), hyperactivity/inattention (scores: 7–10), peer relationship problems (score > 5) will be excluded from the ProWISE-TIP trial and included in SP1 of the ProHEAD consortium. Children and adolescents with a negative screening result for risky alcohol use (CRAFFT-d score < 2) will be included in SPs 2, 4 or 5 of the ProHEAD consortium, depending on which inclusion criteria they meet. Study participants fulfilling inclusion criteria for more than one SP addressing children and adolescents at risk for developing an eating disorder, alcohol-related problems or depressive symptoms (SP2–4) will be allocated randomly to one SP (see CP study protocol [36] for details). There are no restrictions for study participants to take up concomitant care outside ProHEAD.

### Recruitment and randomisation

Participants for the trial will be recruited through the ProHEAD infrastructure with five centres (Heidelberg, Hamburg, Leipzig, Marburg, Schwäbisch Gmünd) actively recruiting and drawing children and adolescents from a national school-based sample. For a detailed description of the recruitment process, please refer to Kaess et al. [36]. After enrolment in the central project, study participants will be allocated to one of the five SPs according to their screening result (Fig. 1). All participants receive an e-mail containing a link to an encrypted webpage where they can register to the respective SP. Participants with a positive screening for at-risk alcohol use are invited to take part in the ProWISE-TIP trial and referred to the SP-specific online baseline assessment (Fig. 3). After completion of the assessment, participants will be randomised to one of the four study arms (A) ProWISE plus TIP for 12 weeks (ProWISE-TIP); (B) ProWISE plus TA for 12 weeks (ProWISE-TA); (C) ProWISE intervention only (ProWISE only); (D) web-based psychoeducation on alcohol use in childhood and adolescence (control group). Randomisation is conducted automatically by the computer program on the basis of predefined lists and following a permutated block design. Randomisation will be stratified by age and gender. Once participants have completed the trial-specific online baseline assessment, they automatically receive a computer-generated e-mail containing a link to access the online content in their respective trial condition.

### The ProWISE-TIP intervention

#### The ProWISE intervention

The intervention tested in this trial is based on a previously positively evaluated, single-session, web-based, motivational intervention for risky substance use in children and adolescents [24, 43]. For the current study, the intervention was refined in order to address children and adolescents aged ≥ 12 years (previously 16–18 years) and in order to address alcohol use only (previously alcohol and other substance use).

The ProWISE intervention is fully automated and relies on an interactive system to generate individually tailored content. All system-generated information directly refers to the participant’s statements assessed in the first place (e.g. alcohol use, gender, weight, perceptions of peer drinking). Navigation through the program is designed as a dialogue based on motivational interviewing (MI) techniques [44] between the user and the program. The intervention comprises the following six components: (1) feedback on individual drinking patterns with information on associated health and developmental risks; (2) normative feedback on descriptive drinking norms about gender- and age-matched peer drinking levels using graphed comparative information; (3) feedback on blood alcohol concentration (BAC) and associated health and other risks for the reported peak drinking episode; (4) importance and confidence rulers with a short summary and feedback to elicit and strengthen readiness to change and exploration of personal strengths, resources and strategies for goal attainment; (5) decisional balance for selection of personal costs and benefits of current alcohol use and a subsequent graphical display of comparative gains and losses of behaviour change in a balance sheet to illustrate ambivalence; and (6) identification and selection of personal high-risk situations for alcohol use and provision of behavioural strategies, e.g. to resist peer pressure. Completion of the ProWISE intervention takes approximately 20 min.

#### Text-message-initiated individualised prompts (TIPs)

The development of the TIPs will be realised in two phases. In phase 1 we will develop the content of the TIPs. Following the abovementioned EMI approach [30], children and adolescents will receive the prompts querying drinking intentions and providing individualised feedback shortly before high-risk situations for excessive alcohol use, i.e. before weekends, every Thursday at 18:00 h. On days following high-risk situations for drinking, i.e. every Sunday, participants will be prompted to report their actual alcohol consumption and will receive tailored feedback according to the relation of drinking intention and actual consumption.

Based on prior research findings on moderators of brief alcohol intervention efficacy, content of the Thursday TIPs will be individually tailored with respect to gender, age, drinking motives and willingness to set a goal for low-risk drinking or abstinence [45, 46]. Sunday TIPs will be tailored to age, gender, actual drinking [47] and to the degree of attainment of the drinking goal defined in the previous Thursday TIP. TIPs’ content will be informed by data which participants provide: (1) in the school-based online baseline assessment (age, gender, alcohol use); (2) in the SP-specific online assessment following enrolment in the SP (drinking motives); and (3) in the assessments of drinking intentions, willingness to set a drinking goal and actual alcohol use as part of the Thursday and Sunday TIPs.

**Fig. 2:**
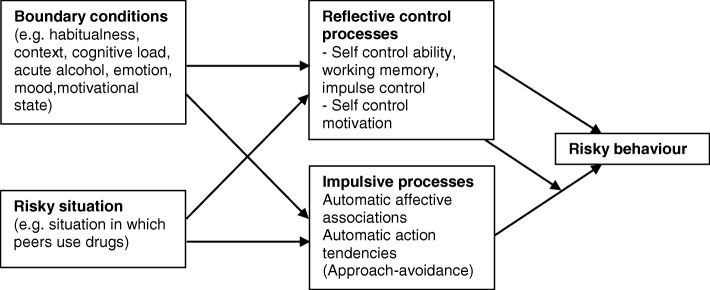
Adapted framework for the prediction of risky behaviour in adolescents (adapted from Wiers et al., 2010; © 2010 Wiers, Ames, Hofmann, Krank and Stacey)

Tailoring of the prompts to participants’ personal drinking motives and risk and consumption profiles is designed to yield high user-centredness, thereby promoting user engagement, a necessary prerequisite for effectiveness of any fully automated intervention [17, 27]. Timing and frequency of the prompts is chosen in order to reach adolescents in the context of their everyday lives, thereby strengthening self-regulatory processes. According to the framework for the prediction of risky behaviour in adolescents [32], risk behaviour is influenced by reflective control processes on the one hand and impulsive processes on the other hand, which, in turn, are influenced by boundary conditions, such as habitualness and motivational state, and characteristics of the situation. The weekly contact with participants before and after potential drinking occasions is supposed to strengthen self-control motivation (through motivational interviewing techniques for eliciting and strengthening motivation to change) and self-control ability (through provision of tailored harm-reduction and drink-less strategies), thereby influencing risky behaviour directly and indirectly (see Fig. 2).

Like the ProWISE intervention, conversational style of the TIPs is designed in an MI congruent way. As central mechanisms of action, the TIPs are designed to increase self-monitoring and promote awareness of potential discrepancies between drinking intentions and actual consumption, thereby raising the awareness of ambivalence as an important mechanism in the development of a motivation to change according to MI theory [44]. According to a recent study, 40% of adolescents and young adults (aged 16–25 years) consume alcohol heavily despite they intent not to [48]. Individualised feedback on this discrepancy embedded in real-life context was found to be effective in reducing heavy drinking [33]. Furthermore, content of the TIPs draws on elements of MI theory by highlighting participants’ responsibility for change and freedom of choice, by providing information on harm-reduction and drink-less strategies as well as by providing feedback on alcohol use and by promoting self-efficacy for the achievement of low-risk alcohol use or abstinence.

Phase 2 of the TIP development will include pilot testing of feasibility and acceptability of the TIPs with *N* = 20 adolescents of the target population of ≥ 12- year-olds with risky alcohol consumption. Two focus groups (*n* = 5 each) will additionally provide feedback on content and conversational style of the TIPs, which will be adapted accordingly.

### Control conditions

Participants in the control groups will receive either: (1) the ProWISE intervention plus weekly TA of alcohol use for 12 weeks every Sunday (ProWISE-TA); or (2) the ProWISE intervention only; or (3) web-based psychoeducation on alcohol use in childhood and adolescence (control). The psychoeducation control group is included in this study in order to test if findings from the previous evaluation of the ProWISE intervention can be replicated, since the intervention has been refined for the current study. Previously, the ProWISE intervention was tested as a self-help program (1) without school-based screening and referral to intervention, (2) in a different age group (16–18 years), and (3) in a slightly different population of at-risk drinkers (i.e. not excluding children and adolescents with AUDIT scores ≥ 20). The ProWISE-TA condition is included in the study design in order to differentiate effects of the TIPs from assessment reactivity [49].

### Data collection

**Fig. 3:**
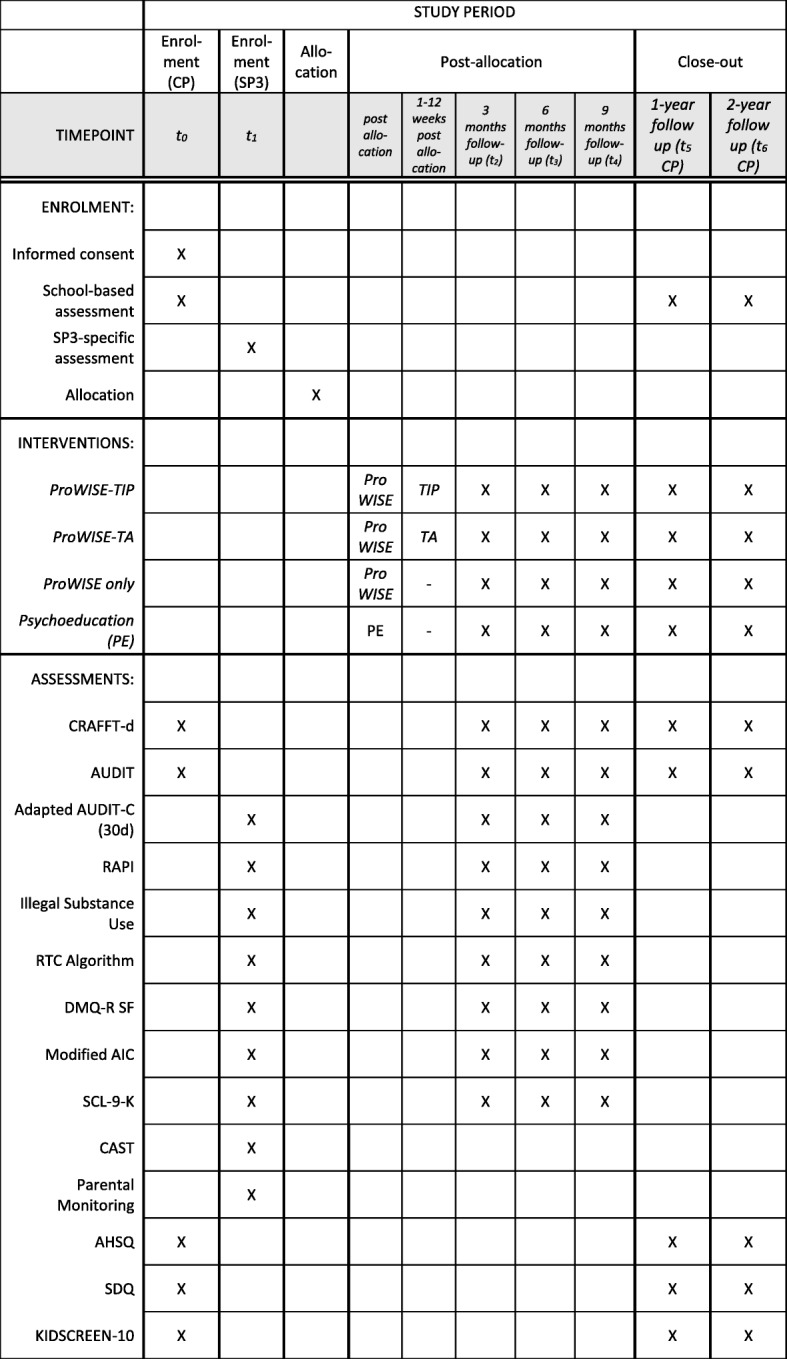
Schedule of enrolment, assessment and intervention. *Note:* *CP* central project, *SP3* sub-project 3, *ProWISE* single-session web-based brief motivational alcohol intervention, *PE* psychoeducation, *TA* text-message-initiated assessment, *TIP* text-message-initiated individualised prompt, *CRAFFT-d* screen for risky alcohol use (Car, Relax, Alone, Forget, Friends, Trouble), *AUDIT* Alcohol Use Disorders Identification Test, *Adapted AUDIT-C (30d)* Alcohol Use Disorders Identification Test - Consumption adapted to assess alcohol use in the past 30 days, *RAPI* Rutgers Alcohol Problem Index, *RTC* Algorithm Readiness to change drinking algorithm, *DMQ-R SF* Drinking Motive Questionnaire-Revised Short Form, *AHSQ* Actual Help-Seeking Questionnaire, *SDQ* Strengths and Difficulties Questionnaire, *KIDSCREEN-10* Quality of life measure for children and adolescents, *CAST* Cannabis Abuse Screening Test, *SCL-9-K* Short version of the Symptom Checklist, *SCS-K-D* German short form of the Self-Control Scale, *AIC* Adolescent Injury Checklist

Participants will complete school-based online assessments at time of enrolment in the CP (t_0_) as well as at one and two years after enrolment (t_5_, t_6_) (Fig. 3). SP-specific assessments will be administered following enrolment in the SP (t_1_) and at the SP-specific follow-ups at three (t_2_), six (t_3_) and nine (t_4_) months after enrolment in SP3. All data will be collected online via central servers that are used for both the school-based assessments and the different interventions that are conducted via the ProHEAD online platform. Data will be handled in accordance with German legal regulations concerning data protection and data security (Data Protection Law of the Federal State of Baden-Wuerttemberg, Data Protection Law of the Free and Hanseatic City of Hamburg and German National Data Protection Laws) as well as EU General Data Protection Regulation. Data storage and transfer will be encrypted. All follow-up assessments will be conducted online via a password-secured website and will be recorded in the individual case report forms (CRF). An independent Data and Safety Monitoring Committee (DSMB) will oversee all aspects of data collection, handling and analysis. The DSMB will comprise independent researchers with expertise in research methodology, child and adolescent mental health, and technology-based alcohol interventions. Members of the DSMB will have their first meeting before inclusion of the first study participant.

### Measures

Sociodemographic data [50] will be assessed as part of the school-based online assessment. Measures assessed for the CP and the other SPs are described elsewhere [36–39]. The CRAFFT-d screening test for risky alcohol use in adolescence in its validated German version [40] will be applied as part of the school-based screening to identify children and adolescents with at-risk alcohol use which will be included in the ProWISE-TIP trial. This six-item questionnaire assesses alcohol-related risk behaviours applying a binary yes/no response format (e.g. ‘Do you ever use alcohol to relax, feel better about yourself, or fit in?’; ‘Do you ever use alcohol while you are by yourself?’). Two or more positive answers indicate risky alcohol use. The CRAFFT-d is validated in the age group of adolescents aged 12–18 years [40].

Drinking motives required for individualisation of the TIPs will be assessed by the Drinking Motive Questionnaire Revised Short Form (DMQ-R SF) [46]. This youth-specific self-report questionnaire comprises 12 items assessing the frequency (never / rarely / sometimes / often / always) of different motives for drinking in the past 12 months (‘In the last 12 months, how often did you drink…’). Three items each represent the four drinking motives enhancement (e.g. ‘…because it’s fun?’), social (e.g. ‘…because it helps you enjoy a party?’), conformity (e.g. ‘…to fit in with a group you like?’) and coping (e.g. ‘...to forget about your problems?’). Scores for drinking motives are calculated by mean sum scores of the items representing the respective drinking motive with higher scores representing more predominant motives. The DMQ-R SF is validated in the age group 12–18 [46].

#### Primary outcome measure

For reasons of comparability, we chose to use the same primary outcome as in our previous study, testing the effectiveness of the original version of the web-based brief alcohol intervention without TIPs [24]. Therefore, the primary outcome is alcohol use in the past 30 days at nine-month follow-up as assessed by an index score for frequency and quantity of drinking and frequency of binge drinking calculated from the first three items of the Alcohol Use Disorders Identification Test (AUDIT-C) [41] adapted to assess drinking in the past 30 days. Frequency of alcohol consumption and frequency of having six drinks at one occasion (binge drinking) are assessed with response options ranging from 0 (never) to 4 (four or more times a week). The assessment of quantity of standard drinks consumed per typical drinking occasion (0 = one or two to 4 = ten or more) is supported by a graphical overview over units of alcoholic drinks defined as standard drinks. The AUDIT-C is validated in a sample of 14- to 18-year-old students in Germany [51].

#### Secondary outcome measures

As secondary outcomes, alcohol-related problems will be assessed by a brief version of the Rutgers Alcohol Problem Index (brief RAPI) [52], a youth-specific measure for alcohol-related problems at the SP-specific follow-ups. Participants are asked 16 questions about the frequency of experiencing different situations in the past three months while they were drinking alcohol or as a result of their alcohol use (e.g. ‘Not able to do your homework or study for a test’; ‘Got into fights with other people (friends, relatives, strangers)’; ‘Wanted to stop drinking but you couldn’t’). Response options range from ‘never’, ‘1–2 times’, ‘3–5 times’, ‘6–10 times’ to ‘more than 10 times’. Higher sum scores indicate more severe alcohol-related problems. The brief RAPI was validated in a sample of 12- to 18-year-olds [52]. Alcohol-related injuries in the past six months will be assessed by a modified version of the Adolescent Injury Checklist (AIC) [53]. Prevalence of experiencing nine different injuries while or shortly after consuming alcohol are assessed (e.g. ‘In the past 6 months, were you injured while or shortly after drinking alcohol … by being in a physical fight with someone? / ... by falling? / ... while riding in a car, truck or bus?’). Response options are ‘yes = 1 / no = 0’ with a higher sum score indicating higher prevalence of alcohol-related injuries in the past six months. Alcohol-related sexually risky behaviour will be recorded by assessing the frequency of experiencing six different risk situations while drinking or shortly after drinking alcohol (e.g. ‘I was sexually harassed’, ‘I had sex, which I couldn’t fully remember later on’). Co-occurring substance use will be assessed as a secondary outcome by the 30-day prevalence of cannabis and other illegal drug use. Additionally, readiness to change alcohol use will be assessed using the Brief Readiness to Change Drinking Algorithm as proposed by Epler et al. [54]. The algorithm comprises three items allowing to categorise risky drinkers’ motivation to change into the stages ‘pre-contemplation’, ‘contemplation’ and ‘action’ according to the Transtheoretical Model of Behaviour Change [55]. Response options for items vary (e.g. ‘Has the amount you drink changed in the past 3 months? Yes, I drink less / Yes, I drink more / No, I drink the same’) and allow allocation of the respondent to one of the three stages of change as described above. The Brief Readiness to Change Drinking Algorithm was validated in an adult population [54].

Additional secondary outcomes will be assessed in the school-based follow-ups at one and two years after enrolment. Help-seeking will be assessed using an adapted version of the Actual Help-Seeking Questionnaire (AHSQ) [56], which assesses actual help-seeking behaviour by listing potential help sources and measuring whether help has been sought from the respective sources within a specified time-period for a specified problem. The 13-item questionnaire comprises three subscales: whether informal help has been sought; whether formal help has been sought; and whether no help has been sought. Response options are 0 = ‘no’, 1 = ‘yes, in the past 12 months’ and 2 = ‘yes, but longer ago then 12 months’. The Actual Help-Seeking Questionnaire (AHSQ) is validated in a sample of 16- to 19-year-old students [56]. Mental health symptoms will be assessed using the Strengths and Difficulties Questionnaire (SDQ), a self-report screening questionnaire for children and adolescents aged 2–17 years [42]. The four subscales (emotional, conduct, hyperactivity and peer problems), each scored on a scale of 0–10, will be assessed. Higher scores indicate a higher level of psychopathology. Quality of life will be assessed using the KIDSCREEN-10 [57]. The KIDSCREEN is an international cross-culturally comparable quality-of-life assessment instrument validated for children and adolescents aged 8–18 years. The KIDSCREEN-10 index comprises 10 items, which provide a global measure of health-related quality of life. Items are answered on a 5-point response scale and are coded in a way that higher values indicate better quality of life. Furthermore, program usage patterns (frequency of replies to TIPs, duration, completion) based on log-file data will be analysed.

#### Further measures

To measure problematic cannabis use in our sample, we will use the Cannabis Abuse Screening Test (CAST) [58]. The CAST consists of one screening item (to evaluate whether cannabis was used in the last year) and six further questions concerning different aspects of cannabis consumption (e.g. if cannabis was already smoked in the morning). A higher sum value in the CAST indicates a more problematic cannabis use. The CAST is validated in a sample of 14- to 22-year-old youth [58]. Additionally, mental wellbeing in our SP sample will be assessed using a short version of the Symptom Checklist (SCL-K-9) [59], which comprises nine items providing a global severity index for general psychopathology. Respondents are asked to indicate how strongly they have suffered from different symptoms in the past seven days. Response options range from 0 = ‘not at all’ to 4 = ‘very strongly’ with higher scores indicating higher levels of psychopathology. The SCL-K-9 is validated in a sample of 14- to 92-year-olds. In order to control for potential moderators and mediators of intervention effectiveness, we will assess dispositional self-control capacity applying the German short form of the Self-Control Scale (SCS-K-D) [60]. The SCS-K-D consists of 13 items with a five-level response format. A higher total score in the SCS-K-D indicates a higher dispositional self-control capacity. The SCS-K-D is validated in a sample of school children attending tenth grade (mean age = 16.6 years). Furthermore, parental monitoring will be assessed by a validated seven-item questionnaire on adolescents’ perceptions of parents’ monitoring comprising the sub-scales parental knowledge (e.g. ‘my parents/guardian know where I am after school’), youth disclosure (e.g. ‘if I am going to be home late, I tell my parents/guardian’), parental solicitation (‘when I go out, my parents/guardian ask me where I’m going’) and parental control (‘when I go out, my parents/guardian tell me what time I’m going to return’) [61]. The parental monitoring scale was developed for and applied in child and adolescent samples [61, 62].

#### Health economic measures

Additionally, cost-effectiveness and cost-utility analyses will be conducted. Data on the cost of interventions will be compared to study outcomes to determine the incremental cost-effectiveness ratio (ICER) of interventions. The ICER is defined as the differential cost of a new treatment and treatment as usual, divided by the outcome differential of the two. Cost-utility analyses will provide information on cost per quality-adjusted life years (QALYs). QALYs are measures combining the additional life years gained by a certain healthcare intervention or program with the quality of life an individual attributes to this lifespan into one single parameter. Thus, QALYs are subjective and universally applicable outcome parameters for comparing health benefits across sectors, disorders, samples or populations. It can be assessed in both patients and healthy individuals. Health-related quality of life will be assessed using the KIDSCREEN-10 [57]. In addition, the health service utilisation of the participants will be assessed by the ‘Mannheimer Modul Ressourcenverbrauch’ (MRV) [63] and transformed into cost estimates for including intervention and treatment as usual costs into the cost-effectiveness and cost-utility analyses of the various study interventions. For transforming health utilisation data, a catalogue of so-called ‘unit costs’ will be compiled for all types of treatments, services or other healthcare measures that were used by study individuals and controls.

### Sample size and power calculation

Two focus groups will be realised in study phase 2 with five participants each. *N* = 20 at-risk alcohol-consuming children and adolescents will be recruited for the pilot testing of the newly designed TIPs. For the RCT, *N* = 15,000 children and adolescents will be assessed for eligibility (ProHEAD school sample). The estimated number of eligible participants for the ProWISE-TIP trial based on current data on the prevalence of risky alcohol consumption is *n* = 3270 [51]. Criteria for the allocation of participants to the five individual ProHEAD trials are based on latest scientific evidence. However, this is the first time that the overall algorithm is applied on a consortium-wide basis simultaneously screening for various mental health problems. Therefore, an intermediate data analysis will be conducted following completion of 10% of the screening assessments (*N* = 1500) in order to determine the actual allocation ratio to the five ProHEAD trials and to adjust the screening algorithm if necessary. Based on prior research, we expect that the ProWISE-TIP intervention reaches a small effect size (f = 0.10) when compared to the ProWISE-only condition and a medium effect size (f = 0.25) when compared to the psychoeducation control group [33, 35]. Thus, a total sample size of *N* = 1076 (intention-to-treat) (*n* = 269 in each trial arm) are needed (power = 0.80; alpha = 0.05, two-tailed; f = 0.10) in order to show superiority of the ProWISE-TIP intervention.

### Statistical analysis

Focus groups in study phase 1 will be evaluated by qualitative content analysis of data gained from the two focus groups. Details on study population will be provided by descriptive data analysis. In the RCT, intention to treat (ITT) analyses of primary data will be based on the available clinical data from all randomised participants after the nine-month follow-up. Missing data will be replaced by multiple imputations. For the primary endpoint, a mixed linear repeated measurement model (LMM) with the participant ID as random, group and time as fixed factor will be performed which is more robust against drop-outs than models without random factor [64] and uses the direct maximum likelihood as the statistical estimation procedure, which results in unbiased estimators under the missing-at-random-assumption [65]. We will use the closed test principle to evaluate differences between the means of groups. An additional analysis will be conducted on the per-protocol sample. The secondary endpoints alcohol-related problems, co-occurring substance use and help-seeking in those with transition to full-threshold alcohol use disorder, other mental health symptoms and quality of life will be examined in an exploratory manner with appropriate procedures, including subgroup analyses of gender of participants. Interim analyses will be performed after each wave of recruitment. Statistical analyses will be carried out with SPSS, Version 22 [66]. Associations between program usage patterns and reductions in alcohol use will be examined using latent class analysis (LCA) with Mplus, Version 5 [67].

### Compliance / Rate of loss to follow-up

According to recent reports on follow-up rates in trials testing web-based alcohol interventions and text-message-based interventions, follow-up data for 80% of randomised individuals are expected at the three-month follow-up, 75% at the six-month follow-up and 70% at the nine-month follow-up (primary endpoint) in the four trial arms [19, 24, 33]. These follow-up rates seem feasible because of the recruitment setting (schools) and the use of incentives. Participants will receive online gift vouchers after completion of each program-specific assessment at the follow-ups at three (€10), six (€20) and nine (€25) months.

### Methods against bias

Proactive recruitment of entire classes in schools will reduce potential selection bias. The Coordination Center for Clinical Trials (KKS) Heidelberg will monitor study-related procedures at the five recruiting centres. Specifically, the recruitment of schools within the target regions and the recruitment of students within these schools will be monitored in order to ensure adherence to the study manual and documentation guidelines as well as equivalent procedures at all sites.

Allocation concealment will be practiced with software support. Validated and standardised measures will be employed. Study participants will be informed about aim and rationale of the study as well as on the randomisation procedure. School-based data collection will be fully automated and web-based, preceded by an in-person introduction to the study and informed consent procedure. SP-specific data assessment will be fully automated and web-based (observer-blind). Weekly TIPs and TAs will be delivered fully automated. To reduce publication bias, the trial was registered in a clinical trials registry (DRKS00014606). Important protocol modifications will be communicated to the registry.

### Dissemination of results

Results of the ProWISE-TIP trial will be published in international peer-reviewed journals and presented on national and international conferences. Additionally, the ProHEAD consortium will disseminate results via the ProHEAD website and the press campaigns accompanying the development of the project. Information regarding the availability of the ProWISE-TIP intervention after the study phase will be communicated to relevant stakeholders, such as schools, youth-specific counselling services and prevention programme providers.

## Discussion

Early onset of alcohol use and excessive alcohol use is a major risk factor for a number of serious negative short-term consequences and for chronification of harmful consumption patterns into adulthood [10]. Therefore, prevention and early intervention addressing risky alcohol use is important to take place at a young age. However, adolescents are hard to reach with prevention measures and they typically show little access to the help system [14–16]. In this trial, we thus aim at developing a user-centred, youth-specific, fully automated, electronic alcohol intervention which has the potential to lower existing barriers for service utilisation and therefore reaches populations of at-risk alcohol-consuming children and adolescents who are often underserved. To our knowledge, this is the first trial to investigate effectiveness and cost-effectiveness of highly individualised, high-frequent booster messages as an add-on to a single-session, web-based, alcohol intervention in the target population of children and adolescents aged ≥ 12 years. The fully automated ProWISE-TIP intervention allows for a standardised delivery of highly tailored content which can potentially be disseminated cost-effectively at a large scale while providing a low-threshold opportunity for service-use, particularly for young non-treatment seeking at-risk populations.

The four-arm trial design will allow evaluating: (1) if the theory-based weekly TIPs following the ProWISE intervention decrease alcohol use among children and adolescents with at-risk drinking patterns significantly stronger compared to psychoeducation only; and (2) if the TIPs add effectiveness to the evidence-based single session ProWISE intervention without additional prompts. Furthermore, the inclusion of the ProWISE-TA condition in the four-arm design of the trial will allow to differentiate effects of the weekly TIP interventions from well documented effects caused by the weekly assessment itself [49].

The results of this study will contribute to the current evidence on user engagement and on effectiveness of web-based alcohol brief interventions with additional boosters for the very young target group of children and adolescents aged ≥ 12 years. The ProWISE-TIP intervention, if proven effective, can be used as a stand-alone youth-specific brief alcohol intervention or as an add-on to future school-based or community-based alcohol prevention programs. The intervention has the potential to be used for early detection and intervention in a variety of settings, such as youth work, schools, party scene, sports clubs or as part of routine paediatric medical care.

### Trial status

The ProWISE-TIP intervention is currently being developed. Recruitment of participants is predicted to start between October 2018 and January 2019. School-based two-year follow-ups are predicted to be completed by March 2021.

## Additional file

Additional file 1: Standard Protocol Items: Recommendations for Interventions Trials (SPIRIT) Checklist. (DOC 123 kb)

## Abbreviations

Adapted AUDIT-C (30d): Alcohol Use Disorders Identification Test - Consumption adapted to assess alcohol use in the past 30 days
AHSQ: Actual Help-Seeking Questionnaire
AIC: Adolescent Injury Checklist
AUDIT: Alcohol Use Disorders Identification Test
BAC: Blood alcohol concentration
CAST: Cannabis Abuse Screening Test
CP: Central project
CRAFFT-d: Screen for risky alcohol use (Car, Relax, Alone, Forget, Friends, Trouble)
CRF: Case report form
DMQ-R SF: Drinking Motive Questionnaire-Revised Short Form
DRKS: German Register for Clinical Trials
EMI: Ecological momentary intervention
ICER: Incremental cost-effectiveness ratio
ITT: Intention to treat
KIDSCREEN-10: Quality of life measure for children and adolescents
LCA: Latent class analysis
LLM: Linear mixed models
MI: Motivational interviewing
MRV: Mannheimer Modul zum Ressourcenverbrauch
PE: Psychoeducation
ProWISE: Prevention of risky alcohol use through Web-based Intervention and SMS-based REminders
QALY: Cost per quality-adjusted life year
RAPI: Rutgers Alcohol Problem Index
RTC Algorithm: Readiness to change drinking algorithm
SBIRT: Substance use screening, brief intervention and referral to treatment
SCL-9-K: Short version of the Symptom Checklist
SCS-K-D: German short form of the Self-Control Scale
SDQ: Strengths and Difficulties Questionnaire
SP: Sub-project
SPIRIT: Standard Protocol Items: Recommendations for Interventions Trials
TA: Text-message-initiated assessment
TIP: Text-message-initiated individualised prompt
